# Heterogeneous *EGFR*, *CDK4*, *MDM4*, and *PDGFRA* Gene Expression Profiles in Primary GBM: No Association with Patient Survival

**DOI:** 10.3390/cancers12010231

**Published:** 2020-01-17

**Authors:** María González-Tablas, Daniel Arandia, María Jara-Acevedo, Álvaro Otero, Ana-Luisa Vital, Carlos Prieto, Nerea González-Garcia, Ana Belén Nieto-Librero, Herminio Tao, Daniel Pascual, Laura Ruiz, Pablo Sousa, Purificación Galindo-Villardón, Alberto Orfao, María Dolores Tabernero

**Affiliations:** 1 Instituto de Investigación Biomédica de Salamanca, IBSAL—University Hospital of Salamanca, 37007 Salamanca, Spain; mtablaspi@usal.es (M.G.-T.); Dan_imed@hotmail.com (D.A.); mariajara@usal.es (M.J.-A.); aoteror@usal.es (Á.O.); cprietos@usal.es (C.P.); nerea_gonzalez_garcia@usal.es (N.G.-G.); ananieto@usal.es (A.B.N.-L.); truehawkmoon@yahoo.es (D.P.); lauraruizmartin22@gmail.com (L.R.); pasoucas@yahoo.es (P.S.); 2 Centre for Cancer Research (CIC-IBMCC, CSIC/USAL, IBSAL) and Department of Medicine, University of Salamanca, 37007 Salamanca, Spain; 3 Neurosurgery Service of the University Hospital of Salamanca, 37007 Salamanca, Spain; 4 Biomedical Research Networking Centre on Cancer–CIBER-CIBERONC, Institute of Health Carlos III, 28029 Madrid, Spain; 5 Sequencing DNA Service (NUCLEUS), University of Salamanca, 37007 Salamanca, Spain; 6 Centre for Neuroscience and Cell Biology and Faculty of Pharmacy, University of Coimbra, 3004-561 Coimbra, Portugal; anavitalcast@gmail.com; 7 Bioinformatics Service (NUCLEUS), University of Salamanca, 37007 Salamanca, Spain; 8 Department of Statistics, University of Salamanca, 37007 Salamanca, Spain; Pgalindo@usal.es; 9 Neurosurgery Service, University Hospital of Coimbra, 3004-561 Coimbra, Portugal; herminiotao@gmail.com; 10 Instituto de Estudios de Ciencias de la Salud de Castilla y León (IECSCYL-IBSAL), 37007 Salamanca, Spain

**Keywords:** glioblastoma, gene expression profile, amplification, intragenic deletions, heterogeneity

## Abstract

Background: The prognostic impact of the expression profile of genes recurrently amplified in glioblastoma multiforme (GBM) remains controversial. Methods: We investigated the RNA gene expression profile of epidermal growth factor receptor (*EGFR*), cyclin-dependent kinase 4 (*CDK4*), murine doble minute 4 (*MDM4*), and platelet derived growth factor receptor alpha (*PDGFRA*) in 83 primary GBM tumors vs. 42 normal brain tissue samples. Interphase FISH (iFISH) analysis for the four genes, together with analysis of intragenic deletions in *EGFR* and *PDGFRA,* were evaluated in parallel at the DNA level. As validation cohort, publicly available RNA gene expression data on 293 samples from 10 different GBM patient series were also studied. Results: At the RNA level, *CDK4* was the most frequently overexpressed gene (90%) followed by *EGFR* (58%) and *PDGFRA* (58%). Chromosome 7 copy number alterations, i.e., trisomy (49%) and polysomy (44%), showed no clear association with *EGFR* gene expression levels. In turn, intragenic *EGFR* deletions were found in 39 patients (47%), including *EGFRvIII* (46%) in association with *EGFRvIVa* (4%), *EGFRvII* (2%) or other *EGFR* deletions (3%) and *PDGFRA* deletion of exons 8–9 was found in only two tumors (2%). Conclusions: Overall, none of the gene expression profiles and/or intragenic *EGFR* deletions showed a significant impact on overall survival of GBM supporting the notion that other still unraveled features of the disease might play a more relevant prognostic role in GBM.

## 1. Introduction

In the last decade multiple genetic alterations have been reported in primary glioblastoma (GBM) [1]. Among those alterations for which a pathogenic and clinical relevance have been recurrently suggested, amplification (i.e., at the DNA level) and/or overexpression (i.e., at the RNA level) of the epidermal growth factor receptor (*EGFR*), cyclin dependent kinase 4 (*CDK4*), mouse double minute 4 (*MDM4*), and platelet-derived growth factor receptor alpha (*PDGFRA*) genes, are included [2,3]. Thus, gene amplification, and particularly *EGFR* gene amplification, is currently considered a major driver of tumor progression with potential prognostic value for risk stratification of GBM [4].

Interestingly, gene amplification has also been associated with copy number alterations (CNA), point mutations, and intragenic deletions of these same four genes. Of note, the intragenic deletions affecting some exons of a gene like the *EGFR* gene, might affect the functional domains of the gene with or without an increase on its expression at the RNA level. An example is the association observed between *EGFR* gene amplification and intragenic deletion of exons 2–7 of *EGFR* (i.e., *EGFRvIIII*)*,* a mutant with potential for targeted therapies. However, the prognostic significance of *EGFR* gene amplification and *EGFRvIII* gene deletion remains controversial [5,6]. In addition to *EGFRvIII,* several other *EGFR* intragenic deletions have been identified which involve different domains of the *EGFR* protein. These include (i) *EGFRvI,* consisting of an exon 1–13 deletion [7]; (ii) *EGFRvII*, an exon 14–15 deletion [8]; (iii) *EGFR vIV* and *EGFR vIVa* [9], both associated with deletion of exons 25–27; (iv) *EGFR vIVb,* consisting of an exon 25–26 deletion [10]; (v) *EGFRvV,* defined by deletion of exons 25–28 [9], and deletions of (vi) exons 2–5 [10]; (vii) exons 12–13 [11]; (viii) exon 4 [12]; (ix) exon 27, and (x) exons 27–28 [13]. In a subset of tumors, two or more of these later *EGFR* deletions coexist and/or are associated with *EGFR* gene amplification [14].

Similar to the *EGFR* gene, the *PDGFRA* gene encoded at chromosome 4q12, is also altered in a subset of GBM tumors that present *PDGFRA* amplification in association with intragenic deletions of exons 8–9 [15]. Other amplified genes in GBM include the *CDK4* and *MDM4* genes encoded at chromosomes 12q14.1 and 1q32.1, respectively [16]. Amplification of these two later genes might be found in association or not with amplification of the *EGFR* gene [4,11]. Altogether, these findings indicate that several gene amplification profiles are present in GBM [4,17], suggesting that gene amplification might play a relevant role in these tumors. Despite the potential impact of gene amplification on the levels of expression of the involved genes, current knowledge about the potential association between these genetic alterations with both the *EGFR*, *PDGFRA*, *MDM4*, and *CDK4* gene expression profiles (GEP) and patient outcome, remains controversial and/or poorly investigated [18,19].

Here we analyzed the relationship between the pattern of expression of the *EGFR*, *CDK4*, *MDM4*, and *PDGFRA* genes, their corresponding CNA profile, and intragenic *EGFR* and *PDGFRA* deletions in 83 GBM tumors vs. 42 normal brain tissue samples. Subsequently, we investigated the potential impact of these GEP and CNA profiles on the outcome of GBM patients. Our findings about the frequency and type of gene amplification and its association with the corresponding GEP were validated in a large cohort of 264 GBM patients and 29 normal brain tissues for whom GEP data was publicly available in the Gene Expression Omnibus (GEO) repository.

## 2. Results

### 2.1. EGFR, CDK4, MDM4, and PDGFRA Gene Expression Levels in GBM vs. Normal Brain Tissues

**Figure 1:**
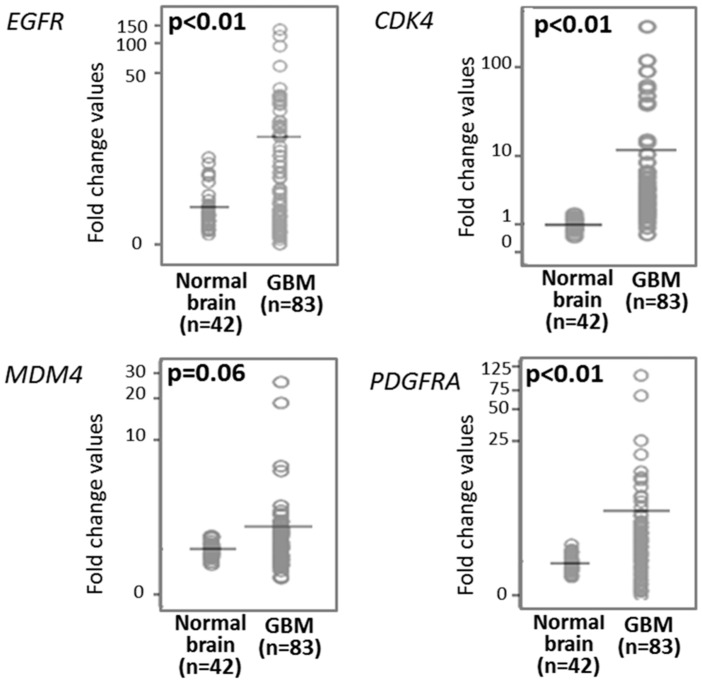
Epidermal growth factor receptor (*EGFR*), cyclin dependent kinase 4 (*CDK4*), mouse double minute 4 (*MDM4*), and platelet-derived growth factor receptor alpha (*PDGFRA*) gene expression levels in GBM (*n* = 83) vs. normal brain tissues (*n* = 42). (Non-parametric comparisons performed using the Mann–Whitney U test). (SPSS 25.0, IBM SPSS, Armonk, NY, USA).

Overall, median gene expression levels for the *EGFR*, *CDK4*, *MDM4*, and *PDGFRA* genes in normal brain tissues was of 0.92, 1.00, 1.01, and 0.96 (FC values), respectively (Table 1). Overall, GBM tumors (*n* = 83) showed higher expression levels for all four genes: 4.11, 3.07, 1.13, and 2.11 FC values for the *EGFR (p* < 0.01)*, CDK4 (p* < 0.01), *MDM4* (*p* = 0.06), and *PDGFRA* (*p* < 0.01) genes, respectively (Table 1 and Figure 1). Higher expression levels for the *EGFR*, *PDGFRA*, *CDK4,* and *MDM4* genes in GBM tumor vs. normal brain tissues was further confirmed in those 264 primary GBM vs. 29 normal brain tissue samples from the GEO database with median FC values of 4.5, 4.4, 2.7, and 2.0 for the *EGFR*, *CDK4*, *MDM4*, and *PDGFRA* genes in the tumor vs. normal brain samples, respectively (Table 1).

**Figure 2:**
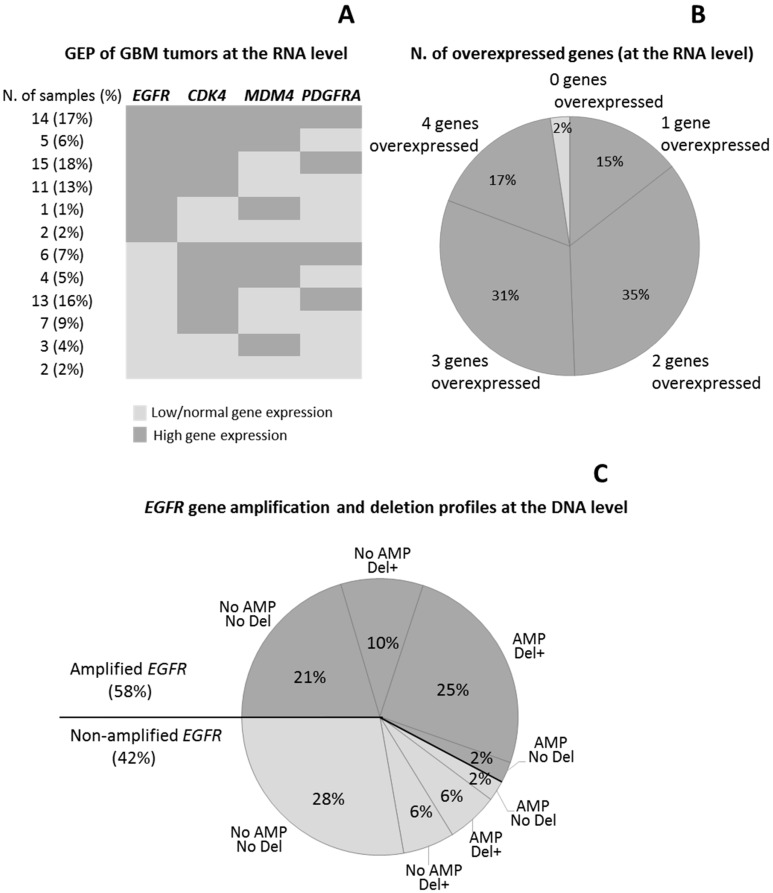
Distribution of GBM cases according to the pattern (**A**) and number (**B**) of overexpressed (*EGFR*, *CDK4*, *MDM4*, and *PDGFRA*) genes, and the association between *EGFR* gene expression levels and the *EGFR* gene deletion/amplification profiles (**C**).

Based on the levels of expression observed for each gene in individual tumor samples vs. normal brain, GBM cases were divided into two groups: (i) GBM patients with low/normal; and (ii) with significantly (*p* < 0.001) higher gene expression levels than those observed in normal brain (Table 2). Among all four genes, *CDK4* was the most frequently overexpressed gene (75/83 GBM tumors; 90%), followed by *EGFR* (58%) and *PDGFRA* (58%), and finally *MDM4* (33/83 cases; 40%) (Figure 2A). Overall, 14/83 tumors (17%) showed simultaneous overexpression of the four genes, 26 (31%) presented with 3/4 overexpressed genes, 29 showed two overexpressed genes (35%), and 12 had only one overexpressed gene (14%) (Figure 2B). Thus, the great majority of our GBM tumors showed amplification of at least one of the four genes investigated, whereas simultaneously low/normal expression levels for the four genes was only found in two samples (2%) (Figure 2A,B).

### 2.2. Association between the EGFR, CDK4, MDM4, and PDGFRA Gene Expression Profiles and Copy Number Alterations

The GEP and CNA pattern for the *EGFR*, *CDK4*, *MDM4*, and *PDGFRA* genes was available in 83/83, 68/83, 68/83, and 68/83 GBM tumor samples evaluated, respectively. Overall, *EGFR* was the most frequently amplified gene at the DNA level (36%), followed by *CDK4* (18%), *MDM4* (9%), and *PDGFRA* (7%) (Table 3). As expected, a clear association was observed between the *EGFR* gene copy number status and expression levels (*p* < 0.001). Thus, a high percentage (*p* = 0.01) of tumors displaying *EGFR* gene amplification showed overexpression of the EGFR gene (77% vs. 23% among non-amplified tumors). In addition, significant differences were found in the median FC values between amplified and non-amplified cases for both the *EGFR* and *MDM4* genes (*p* < 0.001 and *p* = 0.02, respectively) (Table 3). In contrast, no significant association was found between the GEP and CNA status for the *CDK4* and *PDGFRA* genes (Table 3).

### 2.3. Intragenic Deletion and GEP of the EGFR and PDGFRA Genes

*EGFR* intragenic deletions were detected in 39/83 (47%) cases. *EGFRvIII* was found in 38 of these 39 GBM (97%) (Figure 2C), the other case showing an isolated *EGFRvIVa* deletion in the absence of *EGFRvIII*. Of note, in five *EGFRvIII*-mutated GBM, this mutation coexisted with other *EGFR* mutations/deletions in heterozygous: *EGFRvII* in two (2%), *EGFRvIVa* in two (2%), and deletions of exon 25, exons 2–5 and exons 8–28 in one case each; one of these tumors presented simultaneously *EGFRvII*, *EGFRvIII, EGFRvIVa,* and del exons 2–5 (Supplementary Table S3). Overall, *EGFRvIII,* was more frequently (*p* < 0.001) found in tumors carrying *EGFR* amplification (25/38; 66%) than in *EGFR* non-amplified tumors (13/38; 34%) (Figure 2C). Among these *EGFRvIII^+^* cases, *EGFR* gene amplification was associated with chromosome 7 CNA, particularly with trisomy 7 found in 14/38 (37%) cases and other chromosome 7 polysomies detected in another 7/38 tumors (18%) Of note both alterations (trisomy 7 and chromosome 7 polysomies) were also found at lower frequencies 6/38 (16%) and 6/38 cases (16%), respectively (*p* > 0.05) among cases who had no *EGFR* gene amplification (Supplementary Table S3). Deletion of exons 8–9 of the *PDGFRA* gene was detected in only two GBM (2%), one of them showing amplification of the *PDGFRA* and *CDK4* genes.

### 2.4. Impact of EGFR, CDK4, MDM4, and PDGFRA Gene Expression Profiles on Patient Outcome

**Figure 3:**
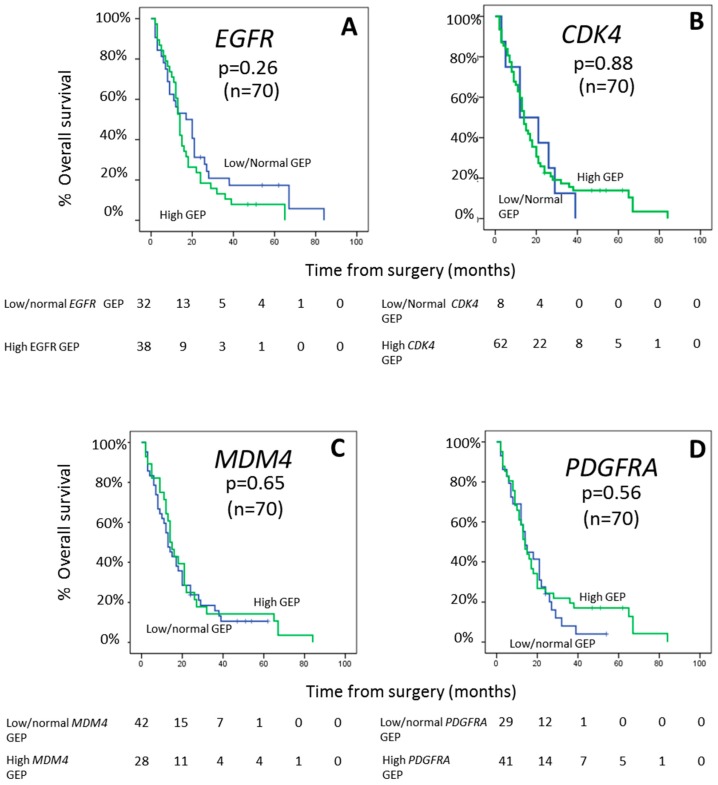
Prognostic impact of *EGFR* (**A**), *CDK4* (**B**), *MDM4* (**C**), and *PDGFRA* (**D**) GEP on overall survival of GBM patients.

The pattern of expression (low/normal vs. high expression) of the *EGFR*, *CDK4*, *MDM4* and *PDGFRA* genes did not show an association with the clinical features of GBM at diagnosis (Supplementary Table S4). No differences in the GEP for the four genes investigated alone or in different combinations among them were observed according to age and sex. In addition, the pattern of expression of the *EGFR*, *CDK4*, *MDM4*, and *PDGFRA* genes, did not show an impact on patient overall survival (*p* > 0.05, respectively) (Figure 3) neither independently nor of the different genes combined. For the *EGFR* gene, this lack of association was further confirmed, also when the presence of intragenic *EGFR* deletions and *EGFR* gene amplification were taken into consideration (Supplementary Figure S1).

## 3. Discussion

The GEP of GBM has been suggested to be of some prognostic value [2,5,6,19,20,21]. Although, controversial results exist in the literature in this regard, and several studies show no predictive value for GEP analyses in GBM [22,23]. Such discrepancies might be, at least in part, due to the heterogeneity of the genetic mechanisms underlying the distinct GEP, including e.g., amplification and mutations of the overexpressed genes. To the best of our knowledge, no study has been reported so far in the literature in which detailed analyses of the GEP together with the most common genetic alterations of the most frequently deregulated genes have been simultaneously investigated in GBM.

Here we investigated for the first time the gene expression profile of four genes (*EGFR*, *CDK4*, *MDM4*, and *PDGFRA*) most frequently amplified at the DNA level in GBM [4] and its potential association with both underlying gene amplification and/or intragenic deletions, and patient outcome. Overall, our results showed highly variable expression profiles for all four genes investigated, overexpression of one or more of these four genes (vs. normal brain tissues) being observed in virtually every GBM. Of note *CDK4* was the most frequently overexpressed gene, followed by *EGFR* and *PDGFRA*, while *MDM4* was overexpressed in a smaller (less than half) fraction of the patients. Interestingly, however, among cases showing overexpression of these four genes, *EGFR* was that showing the highest expression levels. These results about the GEP of GBM were (fully) confirmed in a larger series of 264 GBM cases from publicly available data. In turn, they are in line with previous studies that have demonstrated the existence of altered but highly heterogeneous gene expression profiles (i.e., overexpression) in primary GBM including other studies in smaller patient series [24,25]. In this regard, it should also be noted that the different cells in the same tumor might have distinct gene mutations, and distinct tumor cell subpopulations can be found in different tumor areas (e.g., in the tumor core and the leading edge) further contributing to the observed inter-tumor heterogeneity.

In recent years, accumulated evidence suggested that amplification of the *EGFR* and other genes might play a critical role in the oncogenesis and clinical behavior of GBM [22,26,27]. Despite this, with the exception of a recent study [28], no clear association has been reported in the literature between the GEP and the genetic alterations of individual genes in GBM [29]. In order to investigate the potential association between overexpression of *EGFR*, *CDK4*, *MDM4*, and *PDGFRA*, subsequent analysis of gene amplification was performed at the DNA level. Overall, amplification of *EGFR* was present in a large proportion of our GBM. In contrast, amplification of the *CDK4, MDM4*, and *PDGFRA* genes was restricted to a smaller fraction of the patients. As might be expected, GBM that showed overexpression of *EGFR* and *MDM4*, more frequently displayed amplification of these genes at the DNA level, but with still a significant number of cases showing overexpression of EGFR and *MDM4* in the absence of gene amplification. In contrast, only a tendency (in the absence of significant statistically association) was observed for an association between overexpression and amplification of the *CDK4* and *PDGFRA* genes. Altogether these results suggest that overexpression of one or more of the four genes investigated (*EGFR*, *CDK4*, *MDM4*, and *PDGFRA*) is a hallmark of GBM, which cannot be fully explained on the basis of genetic amplification of the corresponding genes, even when gains of chromosome 7 (in the absence of *EGFR* gene amplification) were also considered.

Based on these results, we then investigated the potential impact of other genetic alterations (i.e., intragenic deletions/mutations) that are frequently observed in the *EGFR* and *PDGFRA* genes, on the expression profile of both genes at the RNA level. In line with previous observations, our results showed that *EGFR* is the most frequently mutated/partially deleted gene in GBM [22,26,30]. As expected, the majority of cases showing intragenic *EGFR* deletions had the *EGFRvIII* variant, alone or in combination with other *EGFR* gene deletions, in association with *EGFR* gene amplification at the DNA level and *EGFR* (RNA) overexpression. Mechanisms for gene overexpression due to mutated *EGFR* gene in GBM include N/C-terminal deletions and deletions of other exons which lead to an oncogenic EGFR protein in some mutations by keeping in the EGFR protein active conformation with an impact also on the RNA expression level of several other genes. Intragenic deletions of the other three genes investigated were rare and they were restricted to a few cases carrying *PDGFRA* gene mutations/deletions. These observations support a critical role for intragenic *EGFR* gene deletions and *EGFR* gene amplification since cross-talk between the intragenic *EGFRvIII* deleted variant and *EGFR* amplification, leads to constitutive activation of the PI3K-AKT signaling pathway and, which might ultimately contribute to explain malignant transformation in GBM [31], as previously suggested by others [22,30]. The close association found here between overexpression of *EGFR* at the RNA level, *EGFR* gene DNA amplification and *EGFRvIII* is in line with previous data from the literature [22,32,33] although there is also the possibility that the *EGFR* gene is not mutated in the two alleles. However, *EGFR* mutations/deletions in homo or heterozygosity (neither alone nor in combination with *EGFR* gene amplification) could fully explain overexpression of the *EGFR* gene. Therefore, our results suggest that despite overexpression of *EGFR*, *PDGFRA*, *CDK4*, and/or *MDM4* is a hallmark of GBM, increased expression of these genes is not fully explained by underlying genetic amplification and/or mutations/deletions, other mechanisms potentially leading also to activation of these genes in GBM. In this regard, previous studies suggested that deregulation of *EGFR*, might also be associated (e.g., induced) by deregulated expression of other genes, particularly genes that involve the PI3-kinase and Akt signaling pathways, such as *CDK4*, leading to an altered cell proliferation and survival. In line with this hypothesis, Liu et al. have also recently demonstrated a synergistic anti-GBM activity of inhibitors of *EGFR* and *CDK4* [34].

Despite all the above, no clear association was found in our study between the *EGFR*, *CDK4*, *MDM4*, and *PDGFRA* gene expression profile and/or the underlying alterations in these four genes and survival of GBM patients, neither when the GEP of the four genes was separately considered nor when it was investigated in combination. Previous studies suggested an association between *EGFR* overexpression and clinical outcome [35,36], both in younger and older GBM patients [27,37,38]. Likewise, an association between *EGFR* amplification and survival has also been previously documented in large series of primary GBM patients [38,39]. However, while in some series *EGFR* overexpression and/or amplification was associated with poorer outcome [40], in others it emerged as a favorable prognostic factor [2,6]; in line with our results, others [22,23] could not confirm this prognostic impact of *EGFR* gene expression and amplification profiles.

## 4. Material and Methods

### 4.1. Patients and Samples

*EGFR*, *CDK4*, *MDM4*, and *PDGFRA* expression was analyzed in a total of 126 frozen samples from adult patients (≥18 years; 50 males and 33 females) with histopathological WHO diagnosis of primary GBM (WHO grade IV gliomas). Most (83/126) samples were from GBM tumors diagnosed as per the WHO criteria [41] 50 males and 33 females; mean age of 59 ± 14 years (range: 21–84 years) who underwent surgery at diagnosis, either (*n* = 58) at the Neurosurgery Service of the University Hospital of Salamanca (Salamanca, Spain) or (*n* = 25) at the University Hospital of Coimbra (Coimbra, Portugal) [25]. From the 83 patients, 34 (47%) underwent complete tumor resection, 31 patients (42%) had a partial tumor resection, and eight (11%) did not undergo surgery. At diagnosis 12 patients (16%) had a Karnofsky performance status (KPS) < 50, 25 patients (23%) between 60 and 70 KPS, 31 patients (41%) between 80 and 90 KPS, and the remaining seven patients (9%) had a KPS index of 100. All patients received standard (similar) therapy protocols and those who died within the first month after surgery, were excluded from the survival analyses. Imprints of individual fresh tumor tissues from these 83 patients were placed in polylysine slides and stored for 3 h at 4 °C before fixation in methanol/acetic acid 3:1 (vol/vol) for further interphase fluorescence in situ hybridization (iFISH) analysis. Remaining tissue samples from these same cases not required for routine diagnostics were immediately frozen in liquid nitrogen and stored at −80 °C until used. Each sample was obtained after surgical resection of the tumor, from patients who had given their prior informed consent according to the Declaration of Helsinki. The study was approved by the local ethics committees of the two participating institutions: Comité de ética de la investigación con medicamentos (CEIm)_Complejo Hospitalario de Salamanca (PI16/00476).

The remaining 42/126 samples corresponded to non-tumoral normal brain tissue specimens, and they included one commercially available normal brain tumor RNA sample (AM7962; Life Technologies. Carlsbad, CA) and 41 samples from age- and sex-matched healthy donors kindly provided by the Principado de Asturias Biobank (PT17/0015/0023 member of the Spanish National Biobank Network Instituto de Salud Carlos III, Madrid, Spain). The *DKMG*/*EGFRvIII* cell line (CL 01008-CLTH, Celther Polska Laboratory, Le-Perray-en-Yvelines, France) was used as positive control for the *EGFRvIII* gene mutation.

Apart from the above listed samples, data derived from GEP arrays of a total of 293 samples from 10 publicly available case-control series corresponding to 264 GBM patients and 29 normal brain tissues available at the GEO repository, were further analyzed as a validation cohort for the GEP identified for the four genes investigated (Supplementary Table S1) with the HGU133Plus2 Affymetrix microarrays [20,21,42,43,44,45,46,47,48].

### 4.2. Gene Expression Profiling Studies

DNA and RNA samples were extracted from frozen tumor specimens using the QIAamp DNA Mini Kit (QIAGEN, Valencia, CA, USA) and the easy-BLUE^TM^ total RNA extraction kit (iNtRON Biotechnology Inc, Seongnam, South Korea) and RNeasy Mini Kit (QIAGEN), respectively. Subsequently, cDNA was synthesized from total RNA (2 µg in 20 µL) treated with 1 µg DNase I (Sigma-Aldrich-Merck, Kenilworth, NJ, UK) using the High Capacity cDNA Reverse Transcription Kit (Applied Biosystems™, Foster City, CA, USA). cDNA was used for *EGFR*, *PDGFRA*, *CDK4*, and *MDM4* gene expression analysis based on an RQ-PCR assay and the BioMark HD System (Fluidigm, South San Francisco, CA, USA), using predesigned FAM-MGB labeled TaqMan^®^ probes (Thermo-Fisher Scientific, Waltham, MA, USA) for the *EGFR* (hs00193306_m1), *PDGFRA* (hs00998018_m1), *CDK4* (hs00364847_m1), and *MDM4* (Hs00967245_m1) genes (Supplementary Table S2). Two housekeeping genes were employed as internal controls to normalize gene expression with identical results: the TATA-Box Binding Protein (TBP; Hs00427620_m1) and Glyceraldehyde-3-Phosphate Dehydrogenase (*GAPDH*; Hs99999905_m1) genes. Ct values were obtained for each sample and deltaCt values calculated to determine the level of expression in a sample by comparing the Ct of each gene with respect to both housekeeping genes (TBP and GAPDH), Ct mean values for TBP being more similar to the four genes investigated than the GAPDH gene expression levels. RQ-PCR assays were performed with the GE 96.96 Dynamic Array™ integrated fluidic circuit (IFC) following the recommendations of the manufacturer (Fluidigm) and the following steps: (i) thermal mix (2 min at 50 °C, 30 min at 70 °C, and 10 min at 25 °C); (ii) Uracil N-glycosylase (UNG, decontaminate) step (2 min at 50 °C and 10 min at 96.5 °C); and (iii) PCR amplification (40 PCR cycles of denaturation at 96 °C for 15 s and annealing at 60 °C for 1 min). To determine the level of expression of each individual gene, deltaCt (ΔCt) values were calculated based on differences observed between the threshold cycle (Ct) obtained for each target gene minus the Ct corresponding to the TBP housekeeping gene. Fold change (FC) values were also calculated for each target sequence and gene per GBM tumor. Cut-off values used to define high or low gene expression levels for individual genes were based on FC values vs. the median values of control (normal brain tissue) samples.

Data derived from the Human Genome U133Plus2.0 arrays were analyzed using Bioconductor and R-package tools (https://www.R-project.org/). Robust multi-array average (RMA) expression was used for data normalization. Variability due to each individual GEO database was removed using the ComBat procedure included in the sva R-package which shrinks the variance among independent series. Gene symbols for the 54,675 probes investigated were annotated, and those without associated information, as well as those corresponding to Affymetrix control probes, were excluded from further analyses. In contrast, multiple probes corresponding to the same gene were kept in the analysis for a total of 44,723 probe sets corresponding to 21,336 genes. Gene expression data was recorded as log2 expression intensity values and differences in gene expression between GBM and normal brain tissues was expressed as FC values for each gene investigated, where FC > 2 corresponded to increased expression and FC < 2 corresponded to normal or lower gene expression levels in GBM vs normal brain tissue.

### 4.3. Assessment of EGFR and PDGFRA Intragenic Deletions

*EGFRvII*, *EGFRvIII*, *EGFRvIV* deletion and *PDGFRA* deletion of exons 8–9 were all analyzed by RQ-PCR using the BioMark HD System (Fluidigm, South San Francisco, CA, USA) and Custom TaqMan^®^ probes and assays, as described above. For the *EGFRvIII* deletion an RQ-PCR SYBR™Green assay was designed based on primers and a probe that exclusively bind to the *EGFR* gene sequences if there is deletion of exons 2 to 7 (the presence of a non-mutated allele or unmutated cells in the same sample, going thereby undetected) and analyzed in a LightCycler 2.0 thermocycler (Roche Diagnostics GmbH, Mannheim, Germany) [49], while for the identification of the *EGFRvII* and *EGFRvIV* mutations, and *EGFR* deletion of exons 2–5 and exons 12–13, a conventional PCR assay followed by Sanger sequencing was used (ABI prism 3130xl, Applied Biosystems). Custom designed primers and probes used are listed in Supplementary Table S2.

### 4.4. Interphase Fluorescence In Situ Hybridization Studies

The *EGFR*/*CEP7* dual color probe (*n* = 83) and the *PDGFRA* (4q12) tri-color break-appart probe kit (*n* = 40) (Vysis Abbott Molecular Inc., Des Plaines, IL, USA) plus the *CDK4*/*CEP12* probe (*n* = 40) (Cytotest, Rockville, MD, USA) and the *MDM4* (1q32/SE 1) probe (*n* = 40) (Kreatech Biotechnology BV, Amsterdam, The Netherlands) were used for iFISH studies, following previously described methods [50]. iFISH Gene amplification was defined for each of the four genes analyzed, whenever ≥7 fluorescent signals were present; below this cut-off (3–6 fluorescence signals) tumors with three or more copies of a gene were considered to have trisomy and polysomy, respectively.

### 4.5. Other Statistical Analyses

The SPSS software (SPSS 25.0, IBM SPSS, Armonk, NY, USA) was used for further statistical analyses. The X^2^ and Mann–Whitney U tests were used to establish the statistical significance of differences observed between groups for categorical and continuous variables, respectively. Gene expression cut-offs were defined based on 95 percentile gene expression values observed in normal brain tissues. Overall survival curves were plotted by the Kaplan and Meier method and compared using the (two-sided) log-rank test, for GBM patients who survived for >1 month after surgery and that had subsequently died or been followed for ≥18 months (in case of patients that remained alive at the moment of closing this study: 70/83 patients).

## 5. Conclusions

Our results highlight the heterogeneity of *EGFR*, *CDK4*, *MDM4*, and *PDGFRA* gene expression profiles in GBM, which can only be partially explained by underlying gene amplification and/or intragenic deletions, revealing the complexity of the mechanisms involved in overexpression of these genes in individual GBM. Independent of the molecular mechanisms involved, the expression profile of the *EGFR*, *CDK4*, *MDM4*, and *PDGFRA* genes does not show a clear impact on the behavior of the disease and patient outcome.

## Figures and Tables

**Table 1:** Epidermal growth factor receptor (*EGFR*), cyclin dependent kinase 4 (*CDK4*), mouse double minute 4 (*MDM4*), and platelet-derived growth factor receptor alpha (*PDGFRA*) gene expression levels in glioblastoma multiforme (GBM) tissues from our series (assessed by RT-QPCR) and from the Gene Expression Omnibus (GEO) database assessed with the HGU133Plus2 Affymetrix microarray.

Gene	Gene Expression Levels					
	Discovery Cohort				Validation Cohort	
	Normal Brain (*n* = 42)	GBM (*n* = 83)	GBM vs. Normal	*p*-Values	GBM vs. Normal (*n* = 293)	*p*-Values
*EGFR*	0.92	4.11	5.54	<0.01	4.5	<0.001
*CDK4*	1.00	3.07	3.88	<0.01	4.4	<0.001
*MDM4*	1.01	1.13	1.22	0.06	2.7	<0.001
*PDGFRA*	0.96	2.11	1.84	<0.01	2.0	<0.001

Results expressed as FC (fold change) values for RT-QPCR and gene expression arrays in tumor versus normal brain tissue samples studied in parallel in the discovery and the validation cohorts, respectively.

**Table 2:** Distribution of GBM according to the gene expression profiles (GEP) observed for the *EGFR*, *CDK4*, *MDM4*, and *PDGFRA* genes.

GBM Subsets	Gene (FC Cut-Off Value ^a^)			
	*EGFR* (2.84)	*CDK4* (1.46)	*MDM4* (1.45)	*PDGFRA* (1.70)
Low/Normal Gene Expression				
N. of Samples (%)	35 (42%)	8 (9%)	50 (60%)	35 (42%)
FC Values	1.20 (0.06–2.83)	1.18 (0.58–1.42)	0.82 (0.58–1.41)	0.81 (0.04–1.68)
High-Gene Expression				
N. of Samples (%)	48 (58%)	75 (90%)	33 (40%)	48 (58%)
FC Values	17.08	3.10	1.76	3.28
	(2.89–251.9)	(1.55–272.3)	(1.46–25.23)	(1.71–102.5)
*p*-Value *	<0.001	<0.001	<0.001	<0.001

^a^ FC (fold change) cut-off values shown between brackets were set at the 95 percentile values observed in normal brain tissue samples; * Comparison of gene expression levels by the Mann–Whitney U test. Results expressed as number (percentage) of cases or as median (range) FC values.

**Table 3:** Association between the copy number (CN) status and gene expression profile of the *EGFR*, *CDK4*, *MDM4*, and *PDGFRA* genes in GBM.

CN Status (N. Cases and Percentage)			Gene Expression Profile of GBM		
			Low/Normal Expression	High Expression	*p*-Value
**Amp*****EGFR*** (***n***** = 83)**	**No** **(64%)**	N. of Cases (%)	28 (53%)	25 (47%)	
		FC	1.14 (0.22–2.32)	6.93 (2.89–76.74)	<0.001
	**Yes** (36%)	N. of Cases (%)	7 (23%)	23 (77%)	
		FC	1.67 (0.06–2.83)	46.02 (7.45–251.94)	<0.001
		*p*-Value	0.10	<0.001	0.01 *
Amp*CDK4* (*n* = 68)	No (82%)	N. of Cases (%)	4 (7%)	52 (93%)	
		FC	1.13 (0.58–1.40)	3.27 (1.55–117.40)	<0.001
	Yes (18%)	N. of Cases (%)	1 (8%)	11 (92%)	
		FC	1.2 (1.2)	10.57 (1.57-89.07)	<0.001
		*p*-Value	1	0.08	0.87 *
Amp*MDM4* (*n* = 68)	No (91%)	N. of cases (%)	38 (61%)	24 (39%)	
		FC	0.86 (0.27–1.41)	1.76 (1.46–5.65)	<0.001
	Yes (9%)	N. of cases (%)	2 (33%)	4 (67%)	
		FC	0.96 (0.55–1.36)	12.09 (1.74–25.23)	<0.001
		*p*-Value	0.85	0.02	0.18 *
Amp*PDGFRA* (*n* = 68)	No (93%)	N. of Cases (%)	26 (41%)	37 (59%)	
		FC	0.63 (0.04–1.55)	3.27 (1.71–66.88)	<0.001
	Yes (7%)	N. of Cases (%)	1 (20%)	4 (80%)	
		FC	0.16 (0.16)	3.34 (2.77–102.47)	<0.001
		*p*-Value	0.25	0.51	0.35 *

CN: copy number; FC: fold change; *: Pearson Chi-Square test for comparison of patient distribution. Results expressed as number of cases (percentage) or as median FC values (range).

## Supplementary Materials

The following are available online at https://www.mdpi.com/2072-6694/12/1/231/s1, Figure S1: Prognostic impact of the GEP of EGFR on overall survival of GBM patients distributed according to the pattern of intragenic *EGFR* deletions and gene expression levels (panel A), and the *EGFR* amplification status (panel B); Table S1: Glioblastoma patient series (*n* = 10) used as validation cohort with publicly available gene expression data in the GEO genomic database (*n* = 293 samples) about tumor (*n* = 264) and normal brain (*n* = 29) tissue samples; Table S2: Probe and primer sequences used to quantify the amount of expression of the *EGFR*, *CDK4*, *MDM4*, and *PDGFRA* genes by RQ-PCR and/or conventional PCR assays; Table S3: GBM tumors displaying *EGFR* amplification and *EGFRvIII* (*n* = 38) deletion and other intragenic deletions of the *EGFR* gene coexisting in the same tumor; Table S4: Relationship between *EGFR*, *CDK4*, *MDM4*, and *PDGFRA* gene expression profiles and the clinical features of the disease at diagnosis.

## References

[1] Crespo I., Vital A.L., Gonzalez-Tablas M., Patino Mdel C., Otero A., Lopes M.C., de Oliveira C., Domingues P., Orfao A., Tabernero M.D. (2015). Molecular and Genomic Alterations in Glioblastoma Multiforme. Am. J. Pathol..

[2] Bienkowski M., Piaskowski S., Stoczynska-Fidelus E., Szybka M., Banaszczyk M., Witusik-Perkowska M., Jesien-Lewandowicz E., Jaskolski D.J., Radomiak-Zaluska A., Jesionek-Kupnicka D. (2013). Screening for EGFR amplifications with a novel method and their significance for the outcome of glioblastoma patients. PLoS ONE.

[3] Szerlip N.J., Pedraza A., Chakravarty D., Azim M., McGuire J., Fang Y., Ozawa T., Holland E.C., Huse J.T., Jhanwar S. (2012). Intratumoral heterogeneity of receptor tyrosine kinases EGFR and PDGFRA amplification in glioblastoma defines subpopulations with distinct growth factor response. Proc. Natl. Acad. Sci. USA.

[4] Gonzalez-Tablas M., Crespo I., Vital A.L., Otero A., Nieto A.B., Sousa P., Patino-Alonso M.C., Corchete L.A., Tao H., Rebelo O. (2018). Prognostic stratification of adult primary glioblastoma multiforme patients based on their tumor gene amplification profiles. Oncotarget.

[5] Montano N., Cenci T., Martini M., D’Alessandris Q.G., Pelacchi F., Ricci-Vitiani L., Maira G., De Maria R., Larocca L.M., Pallini R. (2011). Expression of EGFRvIII in glioblastoma: Prognostic significance revisited. Neoplasia.

[6] Hobbs J., Nikiforova M.N., Fardo D.W., Bortoluzzi S., Cieply K., Hamilton R.L., Horbinski C. (2012). Paradoxical relationship between the degree of EGFR amplification and outcome in glioblastomas. Am. J. Surg. Pathol..

[7] Wong A.J., Ruppert J.M., Bigner S.H., Grzeschik C.H., Humphrey P.A., Bigner D.S., Vogelstein B. (1992). Structural alterations of the epidermal growth factor receptor gene in human gliomas. Proc. Natl. Acad. Sci. USA.

[8] Humphrey P.A., Gangarosa L.M., Wong A.J., Archer G.E., Lund-Johansen M., Bjerkvig R., Laerum O.D., Friedman H.S., Bigner D.D. (1991). Deletion-mutant epidermal growth factor receptor in human gliomas: Effects of type II mutation on receptor function. Biochem. Biophys. Res. Commun..

[9] Ekstrand A.J., Sugawa N., James C.D., Collins V.P. (1992). Amplified and rearranged epidermal growth factor receptor genes in human glioblastomas reveal deletions of sequences encoding portions of the N- and/or C-terminal tails. Proc. Natl. Acad. Sci. USA.

[10] Frederick L., Wang X.Y., Eley G., James C.D. (2000). Diversity and frequency of epidermal growth factor receptor mutations in human glioblastomas. Cancer Res..

[11] Fenstermaker R.A., Ciesielski M.J. (2000). Deletion and tandem duplication of exons 2–7 in the epidermal growth factor receptor gene of a human malignant glioma. Oncogene.

[12] Thorne A.H., Zanca C., Furnari F. (2016). Epidermal growth factor receptor targeting and challenges in glioblastoma. Neuro-Oncology.

[13] Cho J., Pastorino S., Zeng Q., Xu X., Johnson W., Vandenberg S., Verhaak R., Cherniack A.D., Watanabe H., Dutt A. (2011). Glioblastoma-derived epidermal growth factor receptor carboxyl-terminal deletion mutants are transforming and are sensitive to EGFR-directed therapies. Cancer Res..

[14] Johnson B.E., Mazor T., Hong C., Barnes M., Aihara K., McLean C.Y., Fouse S.D., Yamamoto S., Ueda H., Tatsuno K. (2014). Mutational analysis reveals the origin and therapy-driven evolution of recurrent glioma. Science.

[15] Martinho O., Longatto-Filho A., Lambros M.B., Martins A., Pinheiro C., Silva A., Pardal F., Amorim J., Mackay A., Milanezi F. (2009). Expression, mutation and copy number analysis of platelet-derived growth factor receptor A (PDGFRA) and its ligand PDGFA in gliomas. Br. J. Cancer.

[16] Brennan C.W., Verhaak R.G., McKenna A., Campos B., Noushmehr H., Salama S.R., Zheng S., Chakravarty D., Sanborn J.Z., Berman S.H. (2013). The somatic genomic landscape of glioblastoma. Cell.

[17] Furgason J.M., Koncar R.F., Michelhaugh S.K., Sarkar F.H., Mittal S., Sloan A.E., Barnholtz-Sloan J.S., Bahassiel M. (2015). Whole genome sequence analysis links chromothripsis to EGFR, MDM2, MDM4, and CDK4 amplification in glioblastoma. Oncoscience.

[18] Aldape K., Zadeh G., Mansouri S., Reifenberger G., von Deimling A. (2015). Glioblastoma: Pathology, molecular mechanisms and markers. Acta Neuropathol..

[19] Kawaguchi A., Yajima N., Tsuchiya N., Homma J., Sano M., Natsumeda M., Takahashi H., Fujii Y., Kakuma T., Yamanaka R. (2013). Gene expression signature-based prognostic risk score in patients with glioblastoma. Cancer Sci..

[20] Lee Y., Scheck A.C., Cloughesy T.F., Lai A., Dong J., Farooqi H.K., Liau L.M., Horvath S., Mischel P.S., Nelson S.F. (2008). Gene expression analysis of glioblastomas identifies the major molecular basis for the prognostic benefit of younger age. BMC Med. Genom..

[21] Reifenberger G., Weber R.G., Riehmer V., Kaulich K., Willscher E., Wirth H., Gietzelt J., Hentschel B., Westphal M., Simon M. (2014). Molecular characterization of long-term survivors of glioblastoma using genome- and transcriptome-wide profiling. Int. J. Cancer.

[22] Felsberg J., Hentschel B., Kaulich K., Gramatzki D., Zacher A., Malzkorn B., Kamp M., Sabel M., Simon M., Westphal M. (2017). Epidermal Growth Factor Receptor Variant III (EGFRvIII) Positivity in EGFR-Amplified Glioblastomas: Prognostic Role and Comparison between Primary and Recurrent Tumors. Clin. Cancer Res..

[23] Kastenhuber E.R., Huse J.T., Berman S.H., Pedraza A., Zhang J., Suehara Y., Viale A., Cavatore M., Heguy A., Szerlip N. (2014). Quantitative assessment of intragenic receptor tyrosine kinase deletions in primary glioblastomas: Their prevalence and molecular correlates. Acta Neuropathol..

[24] Eskilsson E., Rosland G.V., Solecki G., Wang Q., Harter P.N., Graziani G., Verhaak R.G.W., Winkler F., Bjerkvig R., Miletic H. (2018). EGFR heterogeneity and implications for therapeutic intervention in glioblastoma. Neuro-Oncology.

[25] Vital A.L., Tabernero M.D., Castrillo A., Rebelo O., Tao H., Gomes F., Nieto A.B., Resende Oliveira C., Lopes M.C., Orfao A. (2010). Gene expression profiles of human glioblastomas are associated with both tumor cytogenetics and histopathology. Neuro-Oncology.

[26] van den Bent M.J., Gao Y., Kerkhof M., Kros J.M., Gorlia T., van Zwieten K., Prince J., van Duinen S., Sillevis Smitt P.A., Taphoorn M. (2015). Changes in the EGFR amplification and EGFRvIII expression between paired primary and recurrent glioblastomas. Neuro-Oncology.

[27] Crespo I., Tao H., Nieto A.B., Rebelo O., Domingues P., Vital A.L., Patino Mdel C., Barbosa M., Lopes M.C., Oliveira C.R. (2012). Amplified and homozygously deleted genes in glioblastoma: Impact on gene expression levels. PLoS ONE.

[28] Lassman A.B., Roberts-Rapp L., Sokolova I., Song M., Pestova E., Kular R., Mullen C., Zha Z., Lu X., Gomez E. (2019). Comparison of Biomarker Assays for EGFR: Implications for Precision Medicine in Patients with Glioblastoma. Clin. Cancer Res..

[29] Wang S., Liu F., Wang Y., Fan W., Zhao H., Liu L., Cen C., Jiang X., Sun M., Han P. (2019). Integrated analysis of 34 microarray datasets reveals CBX3 as a diagnostic and prognostic biomarker in glioblastoma. J. Transl. Med..

[30] Maire C.L., Ligon K.L. (2014). Molecular pathologic diagnosis of epidermal growth factor receptor. Neuro-Oncology.

[31] Chakraborty S., Li L., Puliyappadamba V.T., Guo G., Hatanpaa K.J., Mickey B., Souza R.F., Vo P., Herz J., Chen M.R. (2014). Constitutive and ligand-induced EGFR signalling triggers distinct and mutually exclusive downstream signalling networks. Nat. Commun..

[32] Li L., Chakraborty S., Yang C.R., Hatanpaa K.J., Cipher D.J., Puliyappadamba V.T., Rehman A., Jiwani A.J., Mickey B., Madden C. (2014). An EGFR wild type-EGFRvIII-HB-EGF feed-forward loop regulates the activation of EGFRvIII. Oncogene.

[33] Li L., Puliyappadamba V.T., Chakraborty S., Rehman A., Vemireddy V., Saha D., Souza R.F., Hatanpaa K.J., Koduru P., Burma S. (2015). EGFR wild type antagonizes EGFRvIII-mediated activation of Met in glioblastoma. Oncogene.

[34] Liu L., Backlund L.M., Nilsson B.R., Grander D., Ichimura K., Goike H.M., Collins V.P. (2005). Clinical significance of EGFR amplification and the aberrant EGFRvIII transcript in conventionally treated astrocytic gliomas. J. Mol. Med..

[35] Simmons M.L., Lamborn K.R., Takahashi M., Chen P., Israel M.A., Berger M.S., Godfrey T., Nigro J., Prados M., Chang S. (2001). Analysis of complex relationships between age, p53, epidermal growth factor receptor, and survival in glioblastoma patients. Cancer Res..

[36] Wong K.K., Rostomily R., Wong S.T.C. (2019). Prognostic Gene Discovery in Glioblastoma Patients using Deep Learning. Cancers.

[37] Mizoguchi M., Betensky R.A., Batchelor T.T., Bernay D.C., Louis D.N., Nutt C.L. (2006). Activation of STAT3, MAPK, and AKT in malignant astrocytic gliomas: Correlation with EGFR status, tumor grade, and survival. J. Neuropathol. Exp. Neurol..

[38] Houillier C., Lejeune J., Benouaich-Amiel A., Laigle-Donadey F., Criniere E., Mokhtari K., Thillet J., Delattre J.Y., Hoang-Xuan K., Sanson M. (2006). Prognostic impact of molecular markers in a series of 220 primary glioblastomas. Cancer.

[39] Costa B.M., Viana-Pereira M., Fernandes R., Costa S., Linhares P., Vaz R., Pinheiro C., Lima J., Soares P., Silva A. (2011). Impact of EGFR genetic variants on glioma risk and patient outcome. Cancer Epidemiol. Biomark. Prev..

[40] Li J., Liang R., Song C., Xiang Y., Liu Y. (2018). Prognostic significance of epidermal growth factor receptor expression in glioma patients. OncoTargets Ther..

[41] Louis D.N., Perry A., Reifenberger G., von Deimling A., Figarella-Branger D., Cavenee W.K., Ohgaki H., Wiestler O.D., Kleihues P., Ellison D.W. (2016). The 2016 World Health Organization Classification of Tumors of the Central Nervous System: A summary. Acta Neuropathol..

[42] Sun L., Hui A.M., Su Q., Vortmeyer A., Kotliarov Y., Pastorino S., Passaniti A., Menon J., Walling J., Bailey R. (2006). Neuronal and glioma-derived stem cell factor induces angiogenesis within the brain. Cancer Cell.

[43] Murat A., Migliavacca E., Gorlia T., Lambiv W.L., Shay T., Hamou M.F., de Tribolet N., Regli L., Wick W., Kouwenhoven M.C. (2008). Stem cell-related “self-renewal” signature and high epidermal growth factor receptor expression associated with resistance to concomitant chemoradiotherapy in glioblastoma. J. Clin. Oncol..

[44] Wiedemeyer R., Brennan C., Heffernan T.P., Xiao Y., Mahoney J., Protopopov A., Zheng H., Bignell G., Furnari F., Cavenee W.K. (2008). Feedback circuit among INK4 tumor suppressors constrains human glioblastoma development. Cancer Cell.

[45] Grzmil M., Morin P., Lino M.M., Merlo A., Frank S., Wang Y., Moncayo G., Hemmings B.A. (2011). MAP kinase-interacting kinase 1 regulates SMAD2-dependent TGF-beta signaling pathway in human glioblastoma. Cancer Res..

[46] Auvergne R.M., Sim F.J., Wang S., Chandler-Militello D., Burch J., Al Fanek Y., Davis D., Benraiss A., Walter K., Achanta P. (2013). Transcriptional differences between normal and glioma-derived glial progenitor cells identify a core set of dysregulated genes. Cell Rep..

[47] Lu T., Aron L., Zullo J., Pan Y., Kim H., Chen Y., Yang T.H., Kim H.M., Drake D., Liu X.S. (2014). REST and stress resistance in ageing and Alzheimer’s disease. Nature.

[48] Griesinger A.M., Josephson R.J., Donson A.M., Mulcahy Levy J.M., Amani V., Birks D.K., Hoffman L.M., Furtek S.L., Reigan P., Handler M.H. (2015). Interleukin-6/STAT3 Pathway Signaling Drives an Inflammatory Phenotype in Group A Ependymoma. Cancer Immunol. Res..

[49] Yoshimoto K., Dang J., Zhu S., Nathanson D., Huang T., Dumont R., Seligson D.B., Yong W.H., Xiong Z., Rao N. (2008). Development of a real-time RT-PCR assay for detecting EGFRvIII in glioblastoma samples. Clin. Cancer Res..

[50] Vital A.L., Tabernero M.D., Crespo I., Rebelo O., Tao H., Gomes F., Lopes M.C., Orfao A. (2010). Intratumoral patterns of clonal evolution in gliomas. Neurogenetics.

