# Supplementary material for: Heterogeneous EGFR, CDK4, MDM4, and PDGFRA Gene Expression Profiles in Primary GBM: No Association with Patient Survival
Source: Cancers (Basel). 2020 Jan 17;12(1):231. doi: 10.3390/cancers12010231 (PMC7016708; doi:10.3390/cancers12010231)
Supplement: Supplementary file 1 [file cancers-12-00231-s001.zip › cancers-684427-supplementary/SUPPTable1_19NOV.docx]

**Supplementary Table S1**. Glioblastoma patient series (*n = 10*) used as validation cohort with publicly available gene expression data in the GEO genomic data base (*n = 293* samples) about tumor (*n = 264*) and normal brain (*n = 29*) tissue samples.

| **Series Code** | **Type of Sample** | | **Study Reference** |
| --- | --- | --- | --- |
|  | Normal Brain Tissue  (*n = 29*) | GBM Tumor Tissue  (*n = 264*) |  |
| GSE43289 | - | 26*^a^* | Vital A et al. Neuro Oncol 2010^22^ |
| GSE4290 | - | 67 | Sun L et al. Cancer Cell 2006^23^ |
| GSE7696 | - | 68 | Murat J et al. Clin Oncol 2008^24^ |
| GSE9200 | - | 13 | Wiedemeyer R et al. Cancer Cell 2008^25^ |
| GSE13041 | - | 8 | Lee Y et al. BMC Med Genomics 2008^26^ |
| GSE15824 |  | 10 | Grzmil M et al. Cancer Res 2011^27^ |
| GSE29796 | - | 4 | Auvergne R et al. Cell Rep 2013^28^ |
| GSE53733 | - | 68 | Reifenberger G et al. Int J Cancer 2014^29^ |
| GSE53890 | 21 | - | Lu T et al. Nature 2014^30^ |
| GSE66354 | 8 | - | Griesinger AM et al. Immunol Res 2015^31^ |

a: 11 GBM patients from the GSE43289 series were simultaneously evaluated with an SNP-array (GSE42631) and a GEP-array (GSE43289) allowing for direct comparison of tumor copy number status and gene expression data for the *EGFR*, *CDK4*, *MDM4* and *PDGFRA* genes.
