# Supplementary material for: Heterogeneous EGFR, CDK4, MDM4, and PDGFRA Gene Expression Profiles in Primary GBM: No Association with Patient Survival
Source: Cancers (Basel). 2020 Jan 17;12(1):231. doi: 10.3390/cancers12010231 (PMC7016708; doi:10.3390/cancers12010231)
Supplement: Supplementary file 1 [file cancers-12-00231-s001.zip › cancers-684427-supplementary/SUPPTable2_19NOV.docx]

**Supplementary Table S2.** Probe and primer sequences used to quantify the amount of expression of the *EGFR*, *CDK4*, *MDM4* and *PDGFRA* genes by RQ-PCR and/or conventional PCR assays.

| **Gene** | **Targeted Gene Deletion** | **Type of PCR** | **Primers/Probes** |
| --- | --- | --- | --- |
| *EGFR* | *EGFRvIII* | SYBR™Green  RQ-PCR | FW: 5’-GGCTCTGGAGGAAAAGAAAGGTAAT-3’  RV: 5’-TCCTCCATCTCATAGCTGTCG-3’ |
|  |  | TaqMan®  RQ-PCR | FW: 5’-GTCGGGCTCTGGAGGAAAAG-3’  RV: 5’-ATCACGGCTCGTGCGT-3’  TQ:5’-AAAGGTAATTATGTGGTGACAG-3’ |
|  | Exon 2–5 | Conventional PCR | FW: 5‘-GGGCTCTGGAGGAAAAGAAA-3’  RV:5'-AGCAGTCACTGGGGGACTT-3’ |
|  | Exon 12–13 | Conventional PCR | FW: 5’-GACCTCCATGCCTTTGAGAA-3’  RV:5’-TTCTCCACAAACTCCCTTGG-3’ |
|  | *EGFRvII* | Conventional PCR | FW: 5'-GCAACAGAGGTGAAAACAG-3’  RV:5'-TTCGCATGAAGAGGCCGATC-3’ |
|  |  | TaqMan®  RQ-PCR | FW: 5’-GTGGACAAGTGCAACCTTCTG-3’  RV: 5’-GTCTTGAAGGCTGTCCAACGA-3’  TQ: 5’-GAGGGATGCACTGGG-3’ |
|  | *EGFRvIV* | Conventional PCR | FW: 5'-GGGAGTTGATGACCTTTGGA-3’  RV: 5'-AGCTTTGCAGCCCATTTCTA-3’ |
|  |  |  | FW: 5’-CAGCGCTACCTTGTCATTCA-3’  RV:5’-TAATTTGGTGGCTGCCTTTC-3’ |
|  |  | TaqMan®  RQ-PCR | FW: 5’-GTGAGTTGATCATCGAATTCTCCAAAA-3’  RV: 5’-AAAGGCCCGCTGGCT-3’  TQ: 5’-TGTATTCTGAATGACAAGGT-3’ |
|  |  | TaqMan®  RQ-PCR | Hs00193306_m1 |
| *CDK4* | Exon 7–8 | TaqMan®  RQ-PCR | Hs00364847_m1 |
| *MDM4* | Exon 9–10 | TaqMan®  RQ-PCR | Hs00967245_m1 |
| *PDGFRA* | Exon15–16 | TaqMan®  RQ-PCR | Hs00998018_m1 |
|  | Exon 8–9 | TaqMan®  RQ-PCR | FW: 5’-GAGATCACCACTGATGTGGAAAAGA-3’  RV: 5’-AAACTTCCTGGACTATTTTGGCCA-3’  TQ: 5’-CATTATTACATCTTATTTCCTG-3’ |
| *TBP* | Exon 2–3 | TaqMan®  RQ-PCR | Hs00427620_m1 |
| *GAPDH* | Exon 2 | TaqMan®  RQ-PCR | Hs99999905_m1 |

FW: forward primer; RV: reverse primer; TQ: TaqMan probe; RQ-PCR: real-time polymerase chain reaction; NA: not appropriate.
