# Supplementary material for: Heterogeneous EGFR, CDK4, MDM4, and PDGFRA Gene Expression Profiles in Primary GBM: No Association with Patient Survival
Source: Cancers (Basel). 2020 Jan 17;12(1):231. doi: 10.3390/cancers12010231 (PMC7016708; doi:10.3390/cancers12010231)
Supplement: Supplementary file 1 [file cancers-12-00231-s001.zip › cancers-684427-supplementary/SUPPTable3_19NOV.docx]

**Supplementary Table S3**. GBM tumors displaying *EGFR* amplification and *EGFRvIII* (n=38) deletion and other intragenic deletions of the *EGFR* gene coexisting in the same tumor.

| **Sample ID** | ***EGFR* Amplification Status** | **Chr7 Status** | ***EGFRvIII*** | | | | **Other Intragenic Deletions** | | |
| --- | --- | --- | --- | --- | --- | --- | --- | --- | --- |
|  |  |  | SYBR™Green | Sequencing | TaqMan®  Ct/Assay | | Conventional PCR/ Sequencing | TaqMan® | |
| **vIII_1** | **AMP** | **diploid** | + | S | 17.92 | + |  |  | |
| vIII_2 | AMP | diploid | + | S | 25.31 | - |  |  | |
| vIII_3 | AMP | diploid | + | S | 25.50 | - |  |  | |
| vIII_4 | AMP | diploid | NS | NS | 19.33 | + |  |  | |
| vIII_5 | AMP | trisomy | + | S | 12.15 | + | del exon 25 |  | |
| vIII_6 | AMP | trisomy | + | S | 13.62 | + | *EGFRvII* | *EGFRvII* | |
| vIII_7 | AMP | trisomy | + | S | 14.19 | + |  |  | |
| vIII_8 | AMP | trisomy | + | S | 14.27 | + | *EGFRvIVa* | *EGFRvIVa* | |
| vIII_9 | AMP | trisomy | + | S | 14.67 | + |  | | |
| vIII_10 | AMP | trisomy | + | S | 14.71 | + |  | | |
| vIII_11 | AMP | trisomy | + | S | 15.15 | + |  | | |
| vIII_12 | AMP | trisomy | + | S | 16.73 | + |  | | |
| vIII_13 | AMP | trisomy | + | S | 17.23 | + |  | | |
| vIII_14 | AMP | trisomy | + | S | 18.95 | + |  | | |
| vIII_15 | AMP | trisomy | + | S | 23.85 | + |  | | |
| vIII_16 | AMP | trisomy | + | S | 26.35 | - |  | | |
| vIII_17 | AMP | trisomy | + | S | 27.10 | - |  | | |
| vIII_18 | AMP | trisomy | + | S | 25.43 | - |  | | |
| vIII_19 | AMP | polysomy | + | S | 14.40 | + | del exons 2-5  *EGFRvII* | *EGFRvII*  *EGFRvIVa* | |
| vIII_20 | AMP | polysomy | + | S | 13.08 | + |  |  | |
| vIII_21 | AMP | polysomy | + | S | 18.55 | + |  | | |
| vIII_22 | AMP | polysomy | + | S | 24.69 | + |  | | |
| vIII_23 | AMP | polysomy | + | S | 25.19 | - |  | | |
| vIII_24 | AMP | polysomy | + | S | 25.55 | - |  | | |
| vIII_25 | AMP | polysomy | + | S | - | - |  | | |
| vIII_26 | not AMP | diploid | + | S | 25.84 | - |  | | |
| vIII_27 | not AMP | trisomy | + | S | 18.07 | + |  | | |
| vIII_28 | not AMP | trisomy | + | S | 19.75 | + |  | | |
| vIII_29 | not AMP | trisomy | + | S | 20.50 | + |  | | |
| vIII_30 | not AMP | trisomy | + | S | 21.05 | + |  | | |
| vIII_31 | not AMP | trisomy | + | S | 22.69 | + |  | | |
| vIII_32 | not AMP | trisomy | + | S | 25.72 | - |  | | |
| vIII_33 | not AMP | polysomy | + | S | 25.76 | - | del exons 8-28*^a^* | |  |
| vIII_34 | not AMP | polysomy | + | S | 12.44 | + |  | | |
| vIII_35 | not AMP | polysomy | + | S | 17.64 | + |  | | |
| vIII_36 | not AMP | polysomy | + | S | 26.06 | - |  | | |
| vIII_37 | not AMP | polysomy | + | S | - | - |  | | |
| vIII_38 | not AMP | polysomy | - | NS | 24.64 | + |  | | |

Cut-off Ct value for (TaqMan®) detection of *EGFRvIII* was of 24.93 (median + 2SD Ct value for normal brain tissue of 25.92 + 0.99). Ct: Threshold cycle; S: Sequenced; NS: Not sequenced; +: EGFRvIII detected; -: EGFRvIII not detected; del: deletion; *^a^*: Possible deletion after exon 8 extending until the end of the *EGFR* gene.
