# Supplementary material for: Heterogeneous EGFR, CDK4, MDM4, and PDGFRA Gene Expression Profiles in Primary GBM: No Association with Patient Survival
Source: Cancers (Basel). 2020 Jan 17;12(1):231. doi: 10.3390/cancers12010231 (PMC7016708; doi:10.3390/cancers12010231)
Supplement: Supplementary file 1 [file cancers-12-00231-s001.zip › cancers-684427-supplementary/SUPPTable4_19NOV.docx]

**Supplementary Table S4.** Relationship between *EGFR*, *CDK4*, *MDM4* and *PDGFRA* gene expression profiles and the clinical features of the disease at diagnosis.

| **Clinical variables** | | **Gene expression values** | | | | | | | |
| --- | --- | --- | --- | --- | --- | --- | --- | --- | --- |
|  |  | % *EGFR* Over Expression | | *% CDK4* Over Expression | | *% MDM4* Over Expression | | *% PDGFRA* Over Expression | |
|  |  |  | *p-*Value |  | *p-*Value |  | *p-*Value |  | *p-*Value |
| **Gender** | Female (*n = 33*)  Male (*n = 50*) | 58%  58% | 1 | 94%  88% | 0.4 | 48%  34% | 0.3 | 70%  50% | 0.1 |
| Age  (range in years) | 18-45 (*n = 16*) | 69% |  | 94% |  | 25% |  | 56% |  |
|  | 46-65 (*n = 29*) | 62% | 0.4 | 93% | 0.6 | 41% | 0.4 | 62% | 0.8 |
|  | 66-85 (*n = 38*) | 50% |  | 87% |  | 45% |  | 55% |  |
| Tumor location | Frontal (*n = 27*)  Temporal (*n = 28*)  Parietal (*n = 5*)  Occipital (*n = 6*)  Fronto-temporal (*n = 3*)  Fronto-parietal (*n = 2*)  Temporo-parietal (*n = 3*)  Deep (*n = 1*) | 63%  57%  40%  66%  66%  100%  66%  0% | 0.8 | 89%  93%  60%  100%  100%  100%  100%  100% | 0.4 | 37%  32%  60%  50%  0%  50%  33%  100% | 0.7 | 66%  50%  60%  50%  66%  100%  66%  100% | 0.8 |
|  | <50 (*n = 12*) | 66% |  | 100% |  | 42% |  | 66% |  |
| Karnofsky | 60-70 (*n = 25*) | 44% | 0.3 | 88% | 0.2 | 20% | 0.1 | 56% | 0.9 |
| index | 80-90 (*n = 31*) | 68% |  | 94% |  | 52% |  | 61% |  |
|  | 100 (*n = 7*) | 71% |  | 71% |  | 29% |  | 57% |  |
